# Hamartoma of hypothalamus presented as precocious puberty and epilepsy in a 10-year-old girl

**DOI:** 10.1016/j.ijscr.2020.10.065

**Published:** 2020-10-25

**Authors:** Bayar Ahmed Qasim, Ayad Ahmad Mohammed

**Affiliations:** a Department of Medicine, College of Medicine, University of Duhok, Kurdistan Region, Iraq; b Department of Surgery, College of Medicine, University of Duhok, Kurdistan Region, Iraq

**Keywords:** Hamartoma, Hypothalamus, Precocious puberty, Epilepsy, Suprasellar mass

## Abstract

**Background:**

Hamartoma of the hypothalamus represents a well-known but rare cause of central precocious puberty and gelastic epilepsy. Due to the delicate site in which a tumor is located, surgery is often difficult and associated with considerable risks.

**Case presentation:**

10-Year old girl presented with early and regular menstruation at the age of 1 year each cycle lasted for 3 days. She had developed breast, axillary and pubic hair at the age of five, and seven years respectively, with history of difficulty in speech especially articulation and epilepsy since childhood for which she is on medications. She had attacks of an inappropriate laugh. The laboratory tests were consisted with central precocious puberty, MRI shows suprasellar mass. She received leuprolide and antiepileptic medicines until surgery planned. Surgery was done with complete resection, with histopathology showing hypothalamic hamartoma. After surgery, there was complete remission of seizure.

She developed recurrence 4 years later and she is currently on anticonvulsant medications with few attacks of convulsions per week, and she has intellectual disabilities and low school performance.

**Conclusion:**

The treatment of hypothalamic hamartoma associated with generalized epilepsy has been found to improve seizures and behavioral disturbances with an acceptable morbidity rate by using a variety of surgical approaches. Partial resection of a tumor may be sufficient to reduce seizure frequency and to improve behavior and quality of life with few side effects. Best outcomes are achieved when the patients are managed by an experienced multidisciplinary team and lifelong follow up is recommended.

## Highlights

• Hamartoma of the hypothalamus may presents as central precocious puberty and gelastic epilepsy.
• It may be asymptomatic for long period.
• MRI is diagnostic in most of the cases.

## 1 Introduction

Hamartoma of the hypothalamus is a rare congenital malformation of the tuber cinereum, which cause the classical trait of precocious puberty, gelastic seizures, and developmental delay. Precocious puberty may occur in up to one third of patients [[1], [2], [3]].

This condition may be asymptomatic for long period, or may present as precocious puberty and seizures. Seizure in such patients is usually in the form of gelastic seizure and often begins early in life, it is commonly manifested as frequent attacks of inappropriate laughter. Patients later may develop many other types of seizures which eventually become very difficult to treat [1,4].

The diagnosis is usually suspected with the appearance of signs of puberty with other evidences of central nervous system dysfunction [5].

Patients usually have elevated gonadotropin and testosterone levels in the blood. Hypothalamic hamartomas in these patients function autonomously as an accessory hypothalamus [5].

The negative feedback system to the brain from the gonads is intact but it is partially resistant to suppression suggesting that precocious puberty is caused by an autonomous production and release of luteinizing-hormone-releasing factor to the circulation by the vessels which communicate to the pituitary-portal blood system [6].

Affected patients usually have associated behavioral, cognitive disorders, attention deficit disorders and pervasive development which are directly related to the epileptic foci [2].

MRI is diagnostic in most of the cases, it is able to differentiate normal hypothalamic tissue from the hamartomatous tissue. MRI shows a reduction in the density of neurons and a relative gliosis when compared to normal gray matter [7].

Drug resistant seizure is usually treated with microsurgical resection of the tumors. Trans-callosal anterior inter-forniceal approach appears to be the most effective surgical approach with relatively low morbidity and good reduction in the seizure frequency, newer approaches such as endoscopic disconnection or radiosurgery are also used when such facilities are available [3,8].

The work of this report case has been reported in line with the SCARE 2018 criteria [9].

## 2 Patient information

10-year old girl presented with early menstrual cycles. The condition started at age of one year when her parents noticed that their child has developed abnormal vaginal bleeding. Her cycles were regular, each cycle lasted for 3 days.

The patients had a negative drug history, the family history for any relevant genetic information or psychosocial history was negative.

### 2.1 Clinical findings

The patient has well developed breasts, axillary and pubic hair at the age of five, and seven respectively. The parents also gave a history of difficulty in speech especially articulation and abnormal generalized body movements epilepsy since early childhood. She also had attacks of an inappropriate laugh.

### 2.2 Diagnostic assessment

A multidisciplinary team consultation were made and they advised for hormonal assessment and MRI of the brain.

**Fig. 1:**
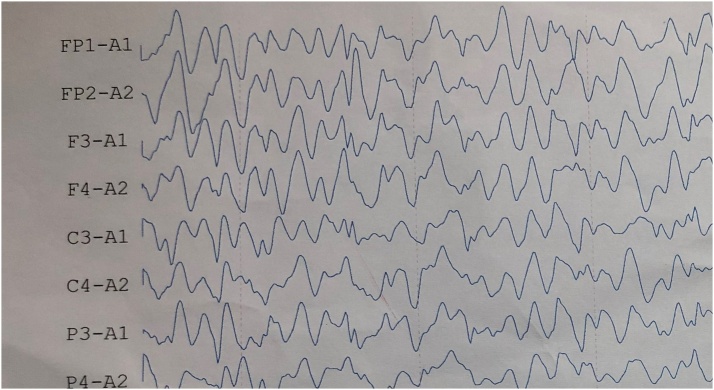
An EEG showing an organized background activity, alpha activity in the anterior leads and beta activity in the posterior leads.

EEG showed an organized background activity, alpha activity in the anterior leads and beta activity in the posterior leads, in responding to opening and closing the eyes, photic stimulations and hyperventilation showed epileptic discharges, with episodes of generalized epilepsy Fig. 1.

Serum TSH levels showed 2.04 u/molL, serum LH 5.49 mlU/mL, serum FSH 4.89 mlU/mL, serum estradiol less than 5 pg/mL, IGF 219 ng/mL, serum cortisol 210 nmol/l, ACTH 10.3 pg/mL, serum prolactin 20 ng/dl, serum parathyroid 25 pg/dl.

**Fig. 2:**
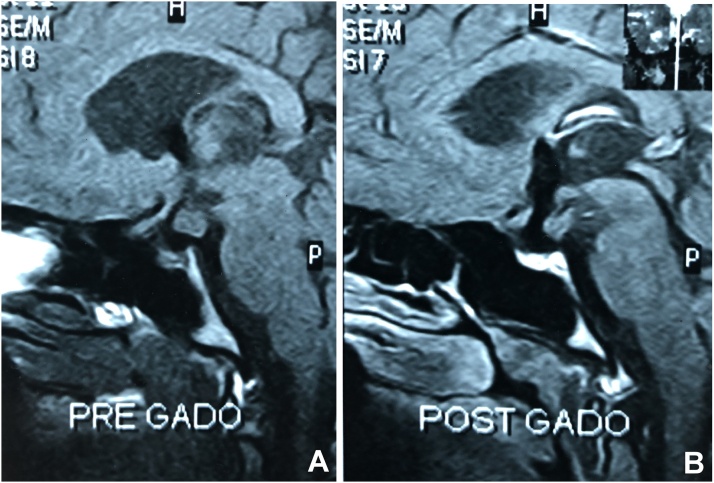
Preoperative MRI of the brain showing an evidence of suprasellar hypothalamic lesion suggestive of hamartoma.

MRI of the brain showed an evidence of 1.5 cm suprasellar right hypothalamic lesion suggestive of hamartoma, Fig. 2.

### 2.3 Therapeutic intervention

The patient received leuprolide and antiepileptic medications and follow-up. During the follow-up period she developed 1 attack of convulsion per week.

The surgical team advised for surgery, during surgery resection of the tumor was performed through trans-callosal anterior inter-forniceal approach and the mass was sent for histo-pathological examination.

The operation was done by a specialist neurosurgeon and the management supervised by a specialist endocrinologist.

Histo-pathological examination showed neurons infiltrating gliotic brain tissue in a very haphazard manner, immunohistochemistry showed a positive staining for GFAP.

**Fig. 3:**
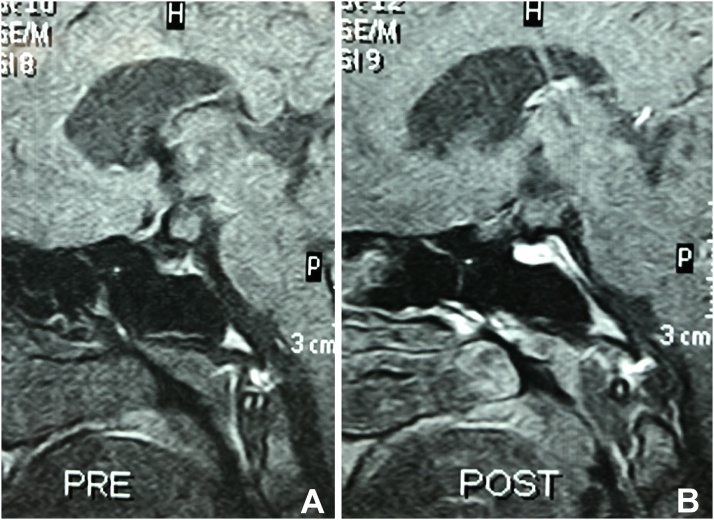
Post-operative MRI of the brain showed an evidence of poorly enhancing lesion in the hypothalamic region suggesting recurrence.

Four years after surgery the patient has attacks of chronic headache with no convulsions, MRI of the brain showed an evidence of poorly enhancing lesion about 6 mm in the hypothalamic region suggesting recurrence Fig. 3.

### 2.4 Follow-up and outcomes

The patient currently is on anticonvulsant medications with few attacks of convulsions per week, and she has intellectual disabilities and low school performance.

## 3 Discussion

Hamartoma of the hypothalamus was considered a very rare finding in the past and was estimated in the past to occur in one person per one million population, however recently with the improvement of the diagnostic methods especially MRI and the better clinical recognition, the incidence appears to be much less [3].

The electroencephalogram (EEG) is often normal and this may result in some delay in the diagnosis. In a proportion of patients the convulsion may progress to generalized spasm [1].

The epilepsy is intrinsically arises within the hamartoma, ablation of the hamartoma by any method results in remission of the seizure activity [2].

Studies shows increased blood flow to the hamartoma during the seizure, but the mechanism of other types of seizures in other foci is not well understood. The pattern of seizure usually changes in adult life and the typical gelastic seizure may not persist [2].

The clinical course of most of the affected individuals is progression of the disease with the development of multiple complex seizures and developmental and mental retardation, however when the patients are appropriately managed surgically, the outcome may be improved [3].

When the facilities for gamma knife resection of the tumor is available, it can be used for resection which usually has good outcomes which may be comparable with the conventional surgery. Open surgery may be associated with significant morbidity [8].

During surgery, the goal should be resection and/or disconnection of the hamartoma from the adjacent normal hypothalamus with preservation of the mammillary bodies, the mammillo-thalamic tracts, the tuber cinereum, and the hypothalamic nuclei [3].

Pathologically, the lesions consist of mature neurons with myelinated and unmyelinated nerve axons, axons are usually arranged in bundles, suggesting a connectivity with the brain tissue. Neurons contain neuro-secretory granules, blood vessels with a fenestrated endothelium and a double basement membrane. Immunofluorescent study shows the presence of luteinizing-hormone-releasing factor in the hamartomatous tissue [2,6].

This clinical condition requires high index of suspicion for the diagnosis, patents may present late when the family is unaware of the symptoms. A detailed history and clinical examination are required, patients may have some other associated anomalites. The best results are obtained when the condition is diagnosed early and the patients should be managed by multidisciplinary team. Close and lifelong follow up is required [3,10].

## Declaration of Competing Interest

The authors report no declarations of interest.

## Funding

None.

## Ethical approval

Ethical approval has been exempted by my institution for reporting this case.

## Consent

An informed written consent was taken from the family for reporting the case and the accompanying images.

## Author contribution

Dr Ayad Ahmad Mohammed and Dr Bayar Ahmed Qasim contributed to the concept of reporting the case and the patient data recording.

Drafting the work, design, and revision done by Dr Ayad Ahmad Mohammed and Dr Bayar Ahmed Qasim.

Final approval of the work to be published was done by Dr Ayad Ahmad Mohammed.

## Registration of research studies

This work is case report and there is no need of registration.

## Guarantor

Dr Ayad Ahmad Mohammed is guarantor for the work.

## Provenance and peer review

Not commissioned, externally peer-reviewed.
